# Perceived Autobiographical Coherence Predicts Depressive Symptoms Over Time Through Positive Self–Concept

**DOI:** 10.3389/fpsyg.2021.625429

**Published:** 2021-03-18

**Authors:** David John Hallford, Jorge Javier Ricarte, Dirk Hermans

**Affiliations:** ^1^School of Psychology, Deakin University, Geelong, VIC, Australia; ^2^Applied Cognitive Psychology Unit Institute of Neurological Disabilities (IDINE), Department of Psychology, School of Education, University of Castilla-La Mancha, Albacete, Spain; ^3^Faculty of Psychology and Educational Sciences, katholieke Universiteit (KU) Leuven, Leuven, Belgium

**Keywords:** narrative coherence, autobiographical coherence, autobiographical memory, self-concept, depressive symptoms, self-efficacy, self-esteem

## Abstract

The coherence of autobiographical memories plays an important role in psychological well-being, as borne out by recent studies. This study aimed to advance this understanding by assessing whether coherence predicted depressive symptoms over time in adults. Further, it aimed to specify mediators through which this association might occur, namely psychological resources of self-esteem self-efficacy, meaning in life, and optimism. A sample of 160 participants (*M* age = 26.4, *SD* = 3.2, 58.1% women) completed surveys at three time-points spaced 1 week apart. The surveys contained measures of the perceived coherence of life stories and autobiographical memories, psychological resources, and depressive symptoms. The results of a path analysis model, controlling for depressive symptoms at baseline, indicated that perceived causal coherence was the only unique predictor of later depressive symptoms, and that this occurred through positive self-concept, represented by self-esteem and self-efficacy. Limitations of the study include no examination of cultural background as a moderating factor and the short time-intervals. Overall, the findings provide further evidence that the perception of how events have unfolded and impacted on one's life and sense of self is particularly important in mitigating depressive symptoms. It extends on our understanding by showing this occurs through changes in self-concept.

## Introduction

Narrative identity refers to the evolving story about one's life and self that is constructed through the comprehension of one's personal experiences. This involves an implicit or explicit process of connecting and integrating personal past experiences (autobiographical memory) to create unity and continuity. These experiences can encompass specific events, recurring or generalized events, and life periods or “chapters.” Fundamental to the integration of experiences is the ability to form coherent accounts of these experiences across one's life. Indeed, coherency in autobiographical thinking is suggested to be a crucial component in developing and maintaining self-identity (Habermas and Bluck, 2000; Adler et al., 2015). As suggested by Habermas and Bluck (2000), this includes knowing when experiences occurred in time (temporal coherence), the causal relationships between them and how change occurred in their life and identity (causal coherence) and recognizing themes about one's life through abstracting experiences and meaning making (thematic coherence). Therefore, coherence is fundamental to narrative content about oneself, such as personal agency, overcoming stressful experiences, and turning-points in life. Some of these themes, as well as coherency itself, have been linked with psychological well-being (e.g., McAdams et al., 2001; Tavernier and Willoughby, 2012; McAdams and McLean, 2013; Liao et al., 2015). Indeed, Waters and Fivush (2015) suggest that having coherent accounts of one's experience may be a critical feature of psychological health and, in particular, foundational to a sense of personal agency.

In support of this, a range of studies have found that more coherent accounts of past experiences are associated with higher levels of psychological well-being (Baerger and McAdams, 1999; Waters and Fivush, 2015; Reese et al., 2017; Lind et al., 2020; Mitchell et al., 2020). In a recent study, self-perceived temporal, causal and thematic coherence and awareness of life stories relating to autobiographical memories were also robustly correlated with psychological resources of self-esteem, self-efficacy, and meaning in life (Hallford and Mellor, 2017). Self-esteem relates to a positive evaluation of one's self-worth, self-efficacy to one's sense of competence and ability to cope with challenges, and meaning in life to a subjective purpose to one's life. These psychological resources are considered to be contributors to overall psychological well-being, and specifically prophylactic against depressive symptoms (Holden, 1992; Paradise and Kernis, 2002; Mascaro and Rosen, 2008; Sowislo and Orth, 2013; Steca et al., 2014). Therefore, they may be mediators that help to explain the association between coherence and well-being. Perceiving past experiences as coherent in terms of when they occurred, their causal association, and their abstractable relatedness may be a key factor in forming perceptions of oneself as have self-worth, competence, and personal meaning. For example, determining one's self-worth might be aided by an ability to abstract positive interpersonal experiences. Viewing oneself as self-efficacious might be more easily done by drawing lines of cause and effect between one's efforts to cope and problem-solve and subsequent outcomes. Lastly, sources of personal meaning might be identified by recognizing commonalities in one's occupational preferences over time. These psychological resources may then mitigate against depressogenic factors including worthlessness and guilt, low self-confidence and hopelessness, and a lack of intrinsic motivation and interest.

However, it is acknowledged that coherency does not necessitate positive interpretations of one's experiences or an increase in psychological resources. Indeed, some studies that have shown coherence is not always directly related to dimensions of well-being, including depression (Hallford and Mellor, 2017; Vanden Poel and Hermans, 2019). Coherence can be related to poorer well-being in the context of traumatic events (Waters et al., 2013). Further, some forms of meaning making may be related to poorer well-being and may be more similar to rumination than to an adaptive resolution (Sales et al., 2013). On rumination, there have been mixed findings in research, with some cross-sectional (Vanaken and Hermans, 2020; Vanderveren et al., 2020a) and prospective studies (Vanderveren et al., 2020b) indicating the association between coherence and well-being might be dependent on rumination, but other findings indicating it is not (Vanderveren et al., 2019). Overall, it can be surmised that having coherent autobiographical memories might not always inherently be adaptive, and there is a need to focus on variables that might help explain the link between coherence and well-being.

Against this background, this study aimed to assess whether perceived autobiographical coherence would predict depressive symptoms over time through psychological resources. In addition to self-esteem, self-efficacy, and meaning in life, we propose that another psychological resource, optimism, might mediate an effect between coherence and depressive symptoms. Optimism refers to the generalized tendency to expect positive outcomes (Carver et al., 2010). It may be that perceiving past experiences as coherently organized and inter-related is a factor in perceiving them as predictable and controllable, and therefore a possible precondition to feeling optimistic (Scheier and Carver, 1985). Further, research shows that optimism increases as a result of reminiscence interventions in which life experiences are interpreted in a coherent and meaningful way, and also leads to reductions in depressive symptoms (Hallford and Mellor, 2016b).

An important distinction between the current study and previous studies is that autobiographical coherence was obtained through self-report of global coherence of memories. Whereas previous research has used coding of written or spoken narratives to determine coherence (e.g., Baerger and McAdams, 1999; Waters and Fivush, 2015; Reese et al., 2017; Lind et al., 2020; Mitchell et al., 2020), the current study used a recently validated measure of subjective perception of awareness of life stories about oneself and autobiographical coherence: The Awareness of Narrative Identity Questionnaire (ANIQ). The ANIQ ask participants to rate their perception of having life stories based on their experiences and their perception of the temporal, causal, and thematic coherence of their autobiographical memories, as inspired by Habermas and Bluck's (2000) proposal that these forms of global coherence are necessary for meaningfully integrating experiences into life stories. There are notable differences between this self-report approach and the coding of coherence from the content of narratives. The ANIQ tries to explicitly capture a person's appraisal of having coherent life stories and the level of perceived coherency that they have with regard to their past experiences. This does not capture narrative identity content and specific themes that one might perceive in their past experiences. Coding written narratives involves a more performative approach in which people provide particular narratives, and each of these is coded for coherence. While this involves explicit storytelling, the assessment of coherence is somewhat implicit, given that coherency is not necessarily the stated purpose of the task. The ANIQ has been found in two separate studies to correlate with the coherence of written narratives of turning-points (mean correlation of *r* = 0.28) and significant relationships (mean correlation of *r* = 0.20) using standardized coding schemes (Hallford and Mellor, 2017; Soroko et al., 2017), therefore showing at least some correspondence between subjective and objectively rated coherence.

It was hypothesized that reported coherence of autobiographical thinking, in terms of temporal, causal, thematic coherence and general awareness of life stories, would predict lower depressive symptoms at a later time, and that this association would occur indirectly through increased psychological resources. No specific hypotheses were made in regard to the types of coherence, given they are all implicated in the development of meaningful autobiographical narratives.

## Methods

### Design

The study used a naturalistic repeated measures design, with three time-points each spaced 1 week apart: T1, T2, and T3. At T1, coherence and depressive symptoms were assessed. At T2, self-esteem, self-efficacy, meaning in life, and optimism were assessed. At T3, depressive symptoms were assessed. A mediation model was tested to assess whether higher coherence at T1 will be associated with less severe depressive symptoms at T3 through the psychological resources at T2 (i.e., higher coherence predicting stronger psychological resources which predicts less severe depressive symptoms). Depressive symptoms at T1 were used as a covariate for psychological resources at T2 and to residualise depressive symptoms at T3.

### Participants

The only inclusion criteria were 18 ≥ years old, English-speaking, and self-identified as Australian. The sample consisted of 160 participants (*M* age = 26.4, *SD* = 3.2, range 18–35; 58.1% women, 41.3% men, and 0.6% non-binary). They were recruited through advertisements on social media and snowballing whereby participants also distributed invitations among their personal networks. As their highest educational attainment, the majority reported having a bachelor degree (47.5%) or postgraduate degree (21.9%), and the remaining had a diploma or certificate (16.3%) or finished high school (14.4%). The majority identified as Caucasian/White European (53.1%), and the remaining as Asian (31.9%), Latino (6.9%), African (5.6%), and “Other ethnicity” (2.5%). Most reported being in paid employment (86.3%), and a quarter were currently studying (26.3%).

With regard to determining sample size, previous research has shown the correlations between the ANIQ and psychological resources to be moderate to strong *r* ≥0.30 (Hallford and Mellor, 2017), and psychological resources and depressive symptoms also moderate to strong over time *r* ≥ 0.29 (Hallford and Mellor, 2016a). Based on these estimates, and using G^*^Power 3.1 (Faul et al., 2007), at least 91 participants would be required at each time-point to detect hypothesized direct effects between the study variables of at least (*r* ≥ 0.29; two-tailed test with an alpha level of 0.05 and power level of 0.80). Oversampling was done to mitigate against a Type II error. The recruited sample size of 160 was adequately powered (0.80) to detect mediation effects (Fritz and MacKinnon, 2007).

### Materials

#### Autobiographical Coherence

The Awareness of Narrative Identity Questionnaire (ANIQ; Hallford and Mellor, 2017), as described above, was used to assess the self-reported awareness that one has life stories about oneself derived from past experiences, and the perceived coherence of autobiographical memories in terms of Habermas and Bluck's (2000) proposed concepts of temporal, causal, and thematic coherence. The ANIQ is a 20-item measure with four, 5-item subscales assessing temporal coherence (e.g., “I can put the events of my life in order of when they occurred”), causal coherence (e.g., “I understand how my life experiences are associated with one another”) and thematic coherence (e.g., “When I recall events and experiences across my lifetime, I can see consistent patterns in the way that I think, feel, and act”), and general awareness of life stories (e.g., “When I think over my life, I can observe how there is a story that tells me who I am”). After respondents are prompted to think generally about their life, they then rate the items on an 11-point, end-defined scale ranging from 0 (completely disagree) to 10 (completely agree). Scores are summed with higher scores indicating higher awareness of life stories and higher perceived coherence. These subscales have shown good psychometric properties, including convergent, divergent, and criterion validity (Hallford and Mellor, 2017). In the current study, the subscales were found to have good internal reliability (Cronbach's α's = 0.89–0.95).

#### Psychological Resources

All psychological resource items were responded to using an 11-point, end-defined scale ranging from 0 (*do not agree at all*) to 10 (*agree completely*), and all scale items were summed with higher scores indicating stronger respective psychological resource. A shortened, five item version of the Rosenberg Self-Esteem Scale (Rosenberg, 2015) was used to assess self-esteem. This short-form scale maintains the measurement fidelity of the full scale, with good convergent and divergent validity, and good internal reliability (Hallford et al., 2013). In the current study, internal reliability was good (Cronbach's α = 0.92). The New General Self-Efficacy Scale (Chen et al., 2001) was used to assess generalized perceptions of competence to deal with stressful situations. The scale consists of eight items and has demonstrated psychometric superiority over other frequently used measures of general self-efficacy (Scherbaum et al., 2006). In the current study, internal reliability was good (Cronbach's α = 0.96). The five-item Presence of Meaning subscale of the Meaning in Life Questionnaire (Steger et al., 2006) was used to assess the extent to which participants felt that their lives were meaningful. This subscale has good psychometric properties and measures meaning in life as a distinct psychological construct (Steger et al., 2006; Hallford et al., 2018). In the current study, internal reliability was good (Cronbach's α = 0.94). Optimism was assessed using a 3-item, shortened version of the psychometrically robust Life Orientation Test – Revised (Scheier and Carver, 1985). This used only the three positively-worded items relating to optimism and excluded the items measuring pessimism and “filler” items. This short version has been shown to operate well in similar research (Hallford et al., 2013). In the current study, internal reliability was good Cronbach's (α = 0.86).

#### Depressive Symptoms

Symptoms of depression were measured using the depression subscale of the short-form Depression, Anxiety, and Stress Scale (Henry and Crawford, 2005). The subscale consists of seven self-report items that are rated on a four-point scale from 0 (*did not apply to me at all*) to 3 (*applied to me very much, or most of the time*) and related to the past week. The items assess core depressive psychopathology of dysphoria, hopelessness, devaluation of life, self-deprecation, lack of interest/involvement, anhedonia, and inertia. The depression subscale possesses very good psychometric properties in the measurement of depressive symptoms (Antony et al., 1998). In the current study the subscale was found to have good internal reliability at T1 (Cronbach's α = 0.91) and T2 (Cronbach's α = 0.92).

### Procedure

Ethics approval was obtained from the University human research ethics committee prior to recruitment commencing. Participants were exposed to the study invitation through an online advertisement. Interested participants clicked on this and were taken to a website that hosted the baseline survey. Here, they were presented with the plain language statement, provided informed consent, and were then prompted to provide demographic information and complete the T1 questionnaire. The baseline measures in this study were completed as part of a larger questionnaire. After completing this all participants completed the T2 survey 1 week later, and then the T3 survey another week later. Thirty-three additional participants expressed interest in the study by visiting the study website, but did not complete the T1 survey. All remaining participants completed all time-points. Participants were prompted up to several times by email and text message to complete the T2 and T3 surveys, which could be completed on mobile phone. No compensation was provided.

### Data Analysis

SPSS 27.0 was used for pre-processing and to generate descriptive statistics and bivariate Pearson correlations. Expectation maximization was used to replace missing responses of items on otherwise complete measures, which was <5% for all variables. Path analysis was used to test the study hypotheses, which allowed for simultaneous tests of direct and indirect associations between the variables. A single step model was conducted with maximum likelihood estimation using AMOS 26.0 software. Bootstrapping with 10,000 bootstrap samples and a bias-corrected and accelerated 95% confidence interval (CI) were used to test for indirect effects. The following fit indices were used to assess the statistical fit of the model to the data: the chi-square value (CMIN) and *p*-value, the relative chi-square statistic (CMIN/df), the root mean square error of approximation (RMSEA), the standardized root mean square residual (SRMR), and the comparative fit index (CFI). We drew on commonly used guidelines provided by Hu and Bentler (1999) to help assess the degree to which the model fit the data: RMSEA ≤ 0.06, SRMR ≤ 0.09, and CFI ≥ 0.95.

## Results

Descriptive statistics and zero-order correlations are shown in Table 1. The ANIQ subscales correlated with one another at T1, as did the psychological sources variables at T2. As anticipated, the ANIQ variables at T1 correlated with psychological resources at T2, and the psychological resources correlated with depressive symptoms at T3. The three ANIQ coherence subscales at T1 also correlated with depressive symptoms at T3. This suggested the possibility of direct and indirect effects in the model. Depressive symptoms at T1 were included as a covariate and as expected were a significant predictor of psychological resources at T2 and depressive symptoms at T3.

**Table 1:** Zero-order correlations between study variables.

	**T1 Depressive** ** symptoms**	**T1 ANIQ** ** Awareness**	**T1ANIQ** ** Temporal**	**T1 ANIQ** ** Causal**	**T1 ANIQ** ** Thematic**	**T2 Self-esteem**	**T2 Self-efficacy**	**T2 Meaning in** ** life**	**T2 Optimism**	**T3 Depressive** ** symptoms**	**Mean (*SD*)**
T1 Depressive symptoms	-										5.3 (5.3)
T1 ANIQ Awareness	−0.14	-									37.4 (9.1)
T1ANIQ Temporal	−0.20^**^	0.48^***^	-								37.4 (10.4)
T1 ANIQ Causal	−0.23^**^	0.66^***^	0.61^***^	-							37.6 (8.1)
T1 ANIQ Thematic	−0.12	0.69^***^	0.53^***^	0.79^***^	-						39.2 (7.8)
T2 Self-esteem	−0.47^***^	0.36^***^	0.34^***^	0.45^***^	0.34^***^	-					37.8 (9.0)
T2 Self-efficacy	−0.38^***^	0.40^***^	0.37^***^	0.47^***^	0.38^***^	0.87^***^	-				60.7 (13.7)
T2 Meaning in life	−0.36^***^	0.41^***^	0.33^***^	0.38^***^	0.31^***^	0.68^***^	0.62^***^	-			34.2 (12.0)
T2 Optimism	−0.34^***^	0.34^***^	0.37^***^	0.37^***^	0.31^***^	0.66^***^	0.69^***^	0.65^***^	-		20.6 (7.2)
T3 Depressive symptoms	0.70^***^	−0.14	−0.20^*^	−0.25^**^	−0.17^*^	−0.59^***^	−0.50^***^	−0.44^***^	−0.40^***^	-	4.1 (4.4)

* *p < 0.05*,

** p < 0.01

*** *p < 0.001. ANIQ, Awareness of Narrative Identity Questionnaire*.

**Figure 1:**
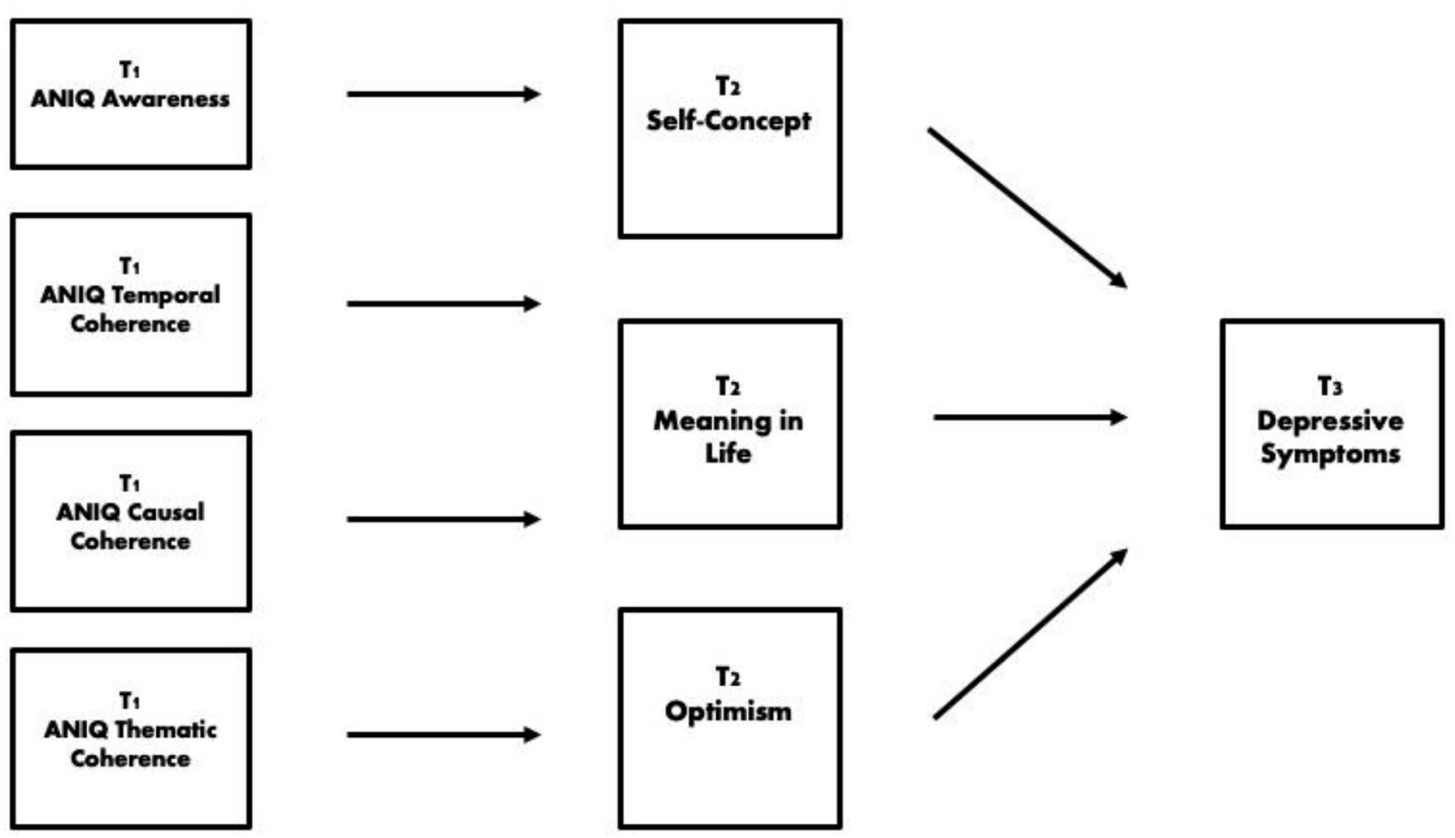
Hypothesized model of indirect effects.

Notably, self-esteem and self-efficacy correlated very highly. Variance inflation factors (VIF) were assessed for all T2 variables as predictors of T3 depressive symptoms. Self-esteem and self-efficacy had elevated VIFs of 5.1 and 5.2 respectively. Given this multicollinearity, and the conceptual overlap between measurement of one's perceived worth and competence, self-esteem and self-efficacy were combined to represent a latent construct of positive self-concept at T2 (see Figure 1 for the final hypothesized model). In contrast, the VIF for meaning in life and optimism were only 2.3 and 2.3, respectively, and they were modeled as originally intended.

### Main Analyses

The results from the model test showed it was a good fit to the data, CMIN = 6.5 (*df* = 4, *p* = 0.161), CMIN/df = 1.6, RMSEA = 0.064, SRMR = 0.054, CFI = 0.99. The model predicted 54% (*p* = 0.007) of variance in depressive symptoms at T3. Consistent with the zero-order correlations, the ANIQ variables correlated significantly with one another at T1 (*r* range 0.45–0.76, *p*'s ≤ 0.001) as did the psychological resources variables (*r* range 0.56–0.64, *p*'s ≤ 0.001). As anticipated as a control variable, depressive symptoms at T1 predicted unique variance in psychological resources at T2 (β range −0.29 to −0.36, *p*'s <0.001) and depressive symptoms at T3 (β = 0.59, *p* = 0.001). The pathways relevant to the study hypotheses are now focused on, but all model parameters are reported in Supplementary Table 1 with confidence intervals and *p-*values.

**Figure 2:**
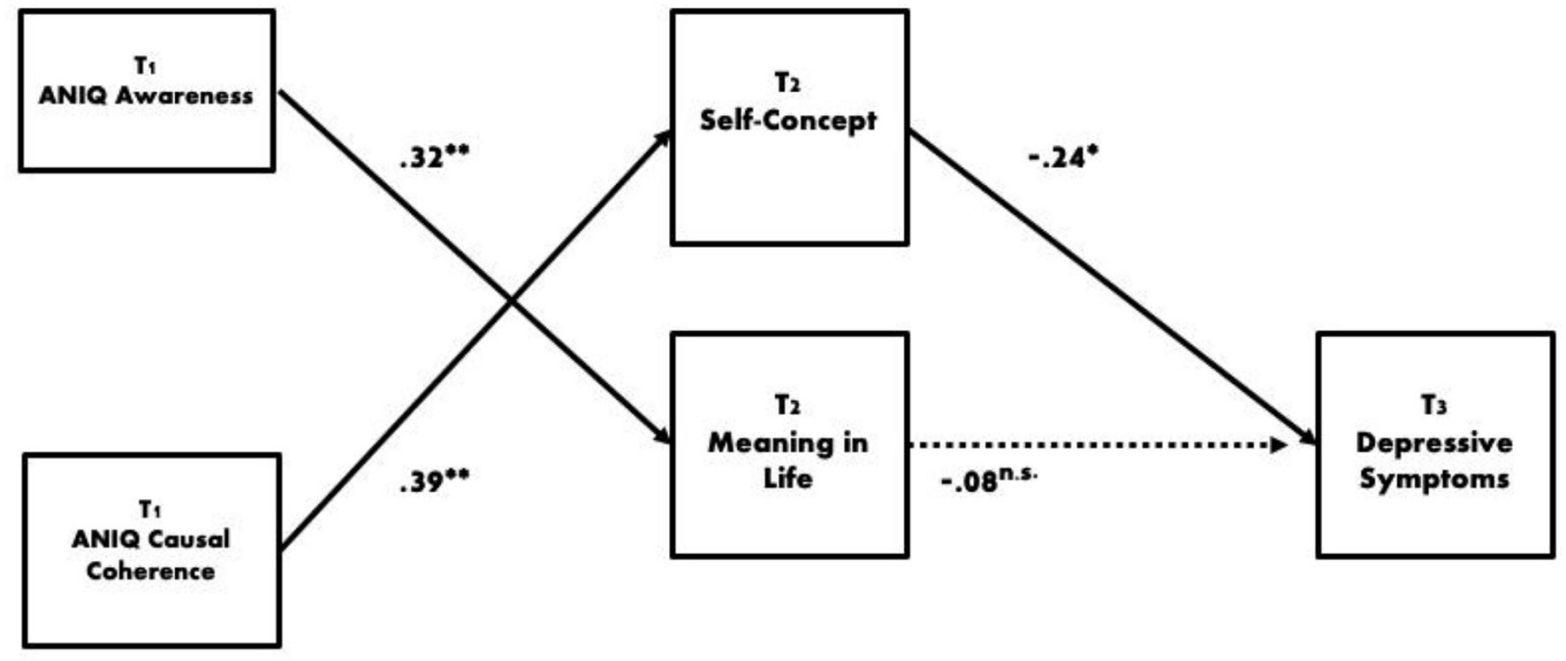
Final model. *p < 0.05, **p < 0.01. ANIQ, Awareness of Narrative Identity Questionnaire. Indirect standardized effect of ANIQ Causal Coherence on Depressive Symptoms = −0.10, CI95% L-0.20 U-0.03, *p* = 0.012.

There were no significant direct pathways between the ANIQ variables at T1 and depressive symptoms at T3 (β range −0.08 to 0.09, *p*'s ≥ 0.242). At T1, the ANIQ variables predicted significant amounts of variance in the psychological resource variables (*R*^2^ = 0.20–34). However, the only independent significant predictors were awareness of narrative identity which predicted meaning in life at T2 (β = 0.32, *p* = 0.001), and causal coherence which predicted self-concept at T2 (β = 0.39, *p* < 0.001). Self-concept at T2 was subsequently the only independent significant predictor of depressive symptoms at T3 (β = −0.24, *p* = 0.008; see Figure 2), with the exception of depressive symptoms at T1; β = 0.59, *p* < 0.001). Tests of indirect effects indicated that higher casual coherence at T1 indirectly predicted lower depressive symptoms at T3 through self-concept (standardized indirect effect = −0.10, CI95% L-0.20 U-0.03, *p* = 0.012). In summary, the ANIQ variables did not directly predict depressive symptoms, but there was an indirect effect of causal coherence on depressive symptoms through a more positive self-concept.

## Discussion

This study was the first to model associations between perceived autobiographical coherence and depressive symptoms over time in adults, and to assess whether these associations occurred through psychological resources. The results supported the hypothesis that coherence would indirectly predict depressive symptoms at a later time-point. Of the types of coherence assessed, only causal coherence indirectly predicted later depression symptoms and only through self-concept. No direct pathways were found between coherence and depressive symptoms.

This finding is consistent with previous cross-sectional research showing correlations between the coherence of autobiographical memories and psychological well-being (Baerger and McAdams, 1999; Waters and Fivush, 2015; Reese et al., 2017; Lind et al., 2020; Mitchell et al., 2020). It also supports findings from recent longitudinal studies showing that higher causal coherence (Mitchell et al., 2020) predicts subsequent depressive symptoms. It extends on those findings by examining these associations over time in an adult sample, assessing additional indices of coherence (i.e., temporal coherence and awareness of life stories), and providing evidence that this relationship might be explained by psychological resources. Given the convergence of the current findings with those using coded narratives (Mitchell et al., 2020), this study provides further evidence that this measure of subjective autobiographical coherence is valid as a means to study narrative coherence and psychological well-being.

The findings provide insight into the process through which causal coherence impacts on psychological well-being. Specifically, that the perception of meaningful causal connections between past experiences might buffer against later depressive symptoms by increasing self-esteem and competence. Causal coherence has been previously shown to foster a sense of personal agency (Adler, 2012). Indeed, qualitative research indicates increased awareness into how past events occurred and how they caused change in one's life is viewed as positively influencing self-understanding (Hallford et al., 2019). Conversely, where life experiences are perceived as fragmented and not meaningfully related it may be more difficult to develop a comprehensible self-concept, increasing susceptible to emotional disorders (Luyten et al., 2020).

Temporal and thematic coherence did not predict unique variance in psychological resources, nor depression, despite having strong zero-order correlations. With regard to temporal coherence, knowing when and in which order autobiographical events happened might be a pre-cursor or pre-requisite to causal coherence (Habermas and Bluck, 2000), and therefore their variance may be largely shared. Indeed, some previous finding support this (Hallford and Mellor, 2017). With regard to thematic coherence, this has been shown to have no association with well-being when causal coherence is concurrently assessed (Mitchell et al., 2020). This suggests that although abstracting and meaning making from experiences might be important in well-being (Tavernier and Willoughby, 2012; Adler et al., 2013), having a strong sense of how events have unfolded and impacted on one's life and sense of self is particularly important for a positive self-concept and in mitigating depressive symptoms. A stronger awareness of life stories did uniquely predict subsequent meaning in life. This subscale assesses perceptions of what could be considered the highest level of abstraction of personal experiences, whereby one perceives that past experiences coalesce into narratives about the kind of person someone is. Despite awareness of these stories explaining unique variance in meaning in life, this did not predict subsequent depressive symptoms. In the current model self-concept was the sole significant predictor of depressive symptoms, however, there may be other well-being outcomes, such as life satisfaction or social or spiritual well-being, that awareness of narrative identity indirectly influences through meaning in life.

Several limitations to this study, its generalisability, and differences with previous research must be noted. Our time-points were spaced very closely together, therefore we cannot be certain of these associations over longer periods. Although previous longitudinal findings (Mitchell et al., 2020) have shown that causal coherence and depressive symptoms are association over a period of years, the mediating role of self-concept over longer periods is less clear. Future studies incorporating longer follow-ups would be useful. In particular longitudinal studies of the period of identify construction from adolescence to adulthood may examine how coherence and self-concept co-develop and potentially mitigate against depressive symptoms. The current study could not effectively assess the influence of culture. This would help clarify how generalisable the findings are. For example, research has indicated that the relationship between episodic detail in autobiographical memory and psychological well-being differs based on culture, with a stronger association for European American's relative to those with an East Asian cultural background (Wang et al., 2018). In that study the authors attributed this stronger association in European American's to the higher valuation of one's autonomous sense of self in Western cultures (Markus and Kitayama, 1991). The current study sampled participants from a Westernized culture, which may explain why self-concept was most strongly predicted by coherence, and why it was the predominant factor in subsequent depressive symptoms. Future cross-cultural research might assess whether culture moderates the observed indirect effects. More broadly, it is possible that non-Westernized countries do not reason about their identity or memories in the way presented in the items on the ANIQ. This is an epistemological question requiring further study. Future research may also measure other socially-related psychological resources, such as social connectedness or perceived ingroup status. Other possibilities are parental reminiscing style, which may moderate the relationship between coherence and positive-concept (Fivush et al., 2011), or attachment style which may have particular relevance in the context of cohesive narratives about relationships and their association with self-concept (Graci and Fivush, 2017). The sample reported fairly low levels of depressive symptoms on average, and therefore the generalizability of these specific findings to clinical samples, or other forms of emotional psychopathology, such as anxiety and stress responses cannot be assumed. Further, given that the sample is predominantly younger adults, the findings may not extend to later periods of the lifespan, in which factor such as meaning in life may be more strongly linked with coherence or depressive symptoms.

In conclusion, this study replicates findings regarding causal coherence as a predictor of depressive symptoms over time. It extends on these by identifying a mediating role of positive self-concept. Some research has indicated the coherence of one's autobiographical thinking is an important factor in change in psychological interventions. In support of that, previous experimental (Hallford and Mellor, 2016c) and treatment outcome studies (Adler and McAdams, 2007; Adler, 2012; Adler et al., 2013) have indicated that autobiographical coherence can change over time as a result of intervention. These findings deepen the understanding of narrative coherence in well-being, and indicate this may occur through the possible mechanism of positive self-concept development.

## Data Availability Statement

The raw data supporting the conclusions of this article will be made available by the authors, without undue reservation.

## Ethics Statement

The studies involving human participants were reviewed and approved by Deakin University Health Ethics Advisory Group. The patients/participants provided their written informed consent to participate in this study.

## Author Contributions

DHa collected and analyzed the data and drafted the manuscript. JR and DHe read and contributed to revisions of the manuscript. All authors contributed to the article and approved the submitted version.

## Conflict of Interest

The authors declare that the research was conducted in the absence of any commercial or financial relationships that could be construed as a potential conflict of interest.
