# Supplementary material for: Perceived Autobiographical Coherence Predicts Depressive Symptoms Over Time Through Positive Self–Concept
Source: Front Psychol. 2021 Mar 18;12:625429. doi: 10.3389/fpsyg.2021.625429 (PMC8044926; doi:10.3389/fpsyg.2021.625429)
Supplement: Supplementary file 1 [file Table_1.docx]

Supplementary Table 1.

*Parameter Estimates for the Path Analysis with 95% Confidence Intervals and p-values*

| **Parameter** | **ß** | **Lower 95% C.I.** | **Upper 95% C.I.** | ***p-*value** |
| --- | --- | --- | --- | --- |
| *Regressions* |  |  |  |  |
| T1 Depressive Symptoms 🡪 T2 Self-Concept | -.36 | -.49 | -.46 | < .001 |
| T1 Depressive Symptoms 🡪 T2 Meaning in Life | -.29 | -.43 | -.17 | < .001 |
| T1 Depressive Symptoms 🡪 T2 Optimism | -.29 | -.43 | -.13 | < .001 |
| T1 Depressive Symptoms 🡪 T3 Depressive Symptoms | .59 | .44 | .70 | .001 |
| T1 ANIQ Awareness 🡪 T2 Self-Concept | .12 | -.09 | .31 | .247 |
| T1 Temporal Coherence 🡪 T2 Self-Concept | -.01 | -.23 | .17 | .834 |
| T1 Causal Coherence 🡪 T2 Self-Concept | .39 | .11 | .63 | .008 |
| T1 Thematic Coherence 🡪 T2 Self-Concept | -.01 | -.28 | .23 | .911 |
| T1 ANIQ Awareness 🡪 T2 Meaning in Life | .32 | .10 | .51 | .004 |
| T1 Temporal Coherence 🡪 T2 Meaning in Life | .04 | -.16 | .22 | .716 |
| T1 Causal Coherence 🡪 T2 Meaning in Life | .20 | -.03 | .45 | .102 |
| T1 Thematic Coherence 🡪 Meaning in Life | -.11 | -.37 | .12 | .352 |
| T1 ANIQ Awareness 🡪 T2 Optimism | .14 | -.07 | .34 | .179 |
| T1 Temporal Coherence 🡪 T2 Optimism | .12 | -.08 | .30 | .229 |
| T1 Causal Coherence 🡪 T2 Optimism | .14 | =.12 | .39 | .289 |
| T1 Thematic Coherence 🡪 T2 Optimism | -.001 | -.29 | .27 | .966 |
| T1 ANIQ Awareness 🡪 T3 Depressive Symptoms | .09 | -.03 | .25 | .162 |
| T1 Temporal Coherence 🡪 T3 Depressive Symptoms | .06 | -.07 | .20 | .368 |
| T1 Causal Coherence 🡪 T3 Depressive Symptoms | -.05 | -.25 | .15 | .602 |
| T1 Thematic Coherence 🡪 T3 Depressive Symptoms | -.08 | -.28 | .12 | .421 |
| T2 Self-Concept 🡪 T3 Depressive Symptoms | -.24 | -.43 | -.04 | .019 |
| T2 Meaning in Life 🡪 T3 Depressive Symptoms | -.08 | -.24 | .09 | .395 |
| T2 Optimism 🡪 T3 Depressive Symptoms | .06 | -.11 | .22 | .476 |
|  | ***r*** | **Lower 95% C.I.** | **Upper 95% C.I.** | ***p-*value** |
| *Correlations* |  |  |  |  |
| T1 ANIQ awareness 🡨🡪 Temporal Coherence | .45 | .28 | .60 | < .001 |
| T1 ANIQ awareness 🡨🡪 Causal Coherence | .64 | .54 | .73 | .001 |
| T1 ANIQ awareness 🡨🡪 Thematic Coherence | .69 | .59 | .76 | .001 |
| T1 Temporal Coherence 🡨🡪 Causal Coherence | .57 | .45 | .68 | < .001 |
| T1 Temporal Coherence 🡨🡪 Thematic Coherence | .50 | .36 | .62 | < .001 |
| T1 Causal Coherence 🡨🡪 Thematic Coherence | .76 | .69 | .82 | .001 |
| T2 Self-Concept 🡨🡪 Meaning in Life | .59 | .47 | .68 | < .001 |
| T2 Self-Concept 🡨🡪 Optimism | .64 | .51 | .73 | < .001 |
| T2 Meaning in Life 🡨🡪 Optimism | .56 | .40 | .68 | .001 |
|  | ***R^2^*** | **Lower 95% C.I.** | **Upper 95% C.I.** | ***p-*value** |
| *Squared Multiple Correlations* |  |  |  |  |
| T2 Self Concept | .34 | .21 | .43 | .006 |
| T2 Meaning in Life | .26 | .13 | .36 | .005 |
| T2 Optimism | .20 | .08 | .29 | .007 |
| T3 Depressive Symptoms | .54 | .41 | .62 | .007 |
